# Relationship between age and bronchodilator response at diagnosis in adult-onset asthma

**DOI:** 10.1186/s12931-020-01441-w

**Published:** 2020-07-13

**Authors:** Minna Tommola, Ha-Kyeong Won, Pinja Ilmarinen, Heewon Jung, Leena E. Tuomisto, Lauri Lehtimäki, Onni Niemelä, Tae-Bum Kim, Hannu Kankaanranta

**Affiliations:** 1 Department of Respiratory Medicine, Seinäjoki Central Hospital, Hanneksenrinne 7, FIN-60220 Seinäjoki, Finland; 2 Department of Internal Medicine, VHS Medical Center, Seoul, South Korea; 3 Department of Allergy and Clinical Immunology, Asan Medical Center, University of Ulsan College of Medicine, 88, Olympic-ro 43-gil, Songpa-gu, Seoul, 05505 South Korea; 4 Allergy Centre, Tampere University Hospital, Tampere, Finland; 5 Faculty of Medicine and Health Technology, Tampere University, Tampere, Finland; 6 Department of Laboratory Medicine and Medical Research Unit, Seinäjoki Central Hospital, Seinäjoki, Finland

**Keywords:** Adult-onset, Asthma, Cohort study, Bronchodilator response, Asthma-COPD overlap, Spirometry

## Abstract

**Background:**

Possible variation in bronchodilator response (BDR) according to age at the diagnosis of adult-onset asthma is unknown. Our aim was to assess if BDR in FEV_1_ is related to age at diagnosis of adult-onset asthma and how many subjects fulfill the 400 mL criterion of BDR, the suggested cut-off for asthma-like reversibility in asthma-COPD overlap (ACO).

**Methods:**

A total of 1030 patients with adult-onset asthma were included; 245 from SAAS (Seinäjoki Adult Asthma Study, Finland) and 785 from COREA (Cohort for Reality and Evolution of Adult Asthma in Korea) cohorts. BDR in FEV_1_ at the diagnosis of asthma was assessed. Patients were divided into groups based on age at asthma diagnosis: < 40, 40–59.9, and ≥ 60 years. The cohorts were analyzed separately.

**Results:**

BDR % in FEV_1_ did not differ between the groups of different age at asthma diagnosis and no correlation between BDR and age was found. Of patients aged ≥40 years, only 18% (SAAS-cohort) and 5% (COREA-cohort) reached the 400 mL BDR in FEV_1_. After exclusion of possible ACO patients, the results remained similar.

**Conclusion:**

By using two large cohorts of steroid-naive patients with asthma, we have shown that BDR at diagnosis of asthma is constant over large age span range, and the limit of 400 mL in BDR in FEV_1_ is rarely reached.

**Trial registration:**

Seinäjoki Adult Asthma Study is registered at ClinicalTrials.gov with identifier number NCT02733016.

## Introduction

Asthma is a chronic, heterogeneous disease, characterized by airway inflammation and variable bronchial obstruction [1]. Reversibility in FEV_1_ of ≥12% and ≥ 200 mL after administration of bronchodilator has been regarded significant, and a key finding when diagnosing asthma [1–3]. However, bronchial reversibility has also been reported in COPD [4, 5], although being usually less than 400 mL in FEV_1_ [6]. Asthma-COPD overlap (ACO) is a novel recognized phenotype of airways diseases concerning adult patients, but little is still known about ACO and its diagnostics [6–9]. Symptomatic evaluation has been suggested and spirometric features such as FEV_1_/FVC < 0.70 and a bronchodilator response of at least 12% or 15% and 400 mL have been proposed to be compatible with a diagnosis of ACO in subjects with sufficient smoking history [6, 10–12]. Recently, it has been suggested that a patient with fixed airway obstruction and smoking history compatible with COPD could be considered to have ACO if he/she has either a high reversibility of obstruction (> 400 mL BDR in FEV_1_) or a diagnosis of asthma before the age of 40 years [7]. The revised criteria for ACO have already been criticized since the majority of asthma has been reported to be diagnosed after 40 years of age in women [13–16], and a BDR of ≥400 mL in FEV_1_ in asthma has been shown to detect predominantly young males [17].

There is, unfortunately, lack of high quality evidence on correct cut-off for BDR to distinguish asthmatics from healthy subjects, and even less is known about the ability of BDR to differentiate between asthma, COPD and ACO [3]. Moreover, smoking among patients with asthma is reported to be nearly as common as among healthy population, up to 26% of asthmatics being smokers [18–20]. This obligates us to pay special attention to the differential diagnostics between asthma, COPD and ACO, especially considering the clinical circumstances where patients have severe symptoms but no previous diagnoses. Previous studies of asthma have usually excluded smoking patients and those with heavy smoking history, and thus, an urgent need for real-life asthma studies including smoking patients has been recognized [6, 20].

In reflection to the proposed criteria of ACO, our aim was to evaluate whether BDR varies with age at diagnosis of adult-onset asthma, and how large proportion of patients fulfil the criterion of 400 mL in BDR, by using data of two, well-described, real-world asthma cohorts.

## Methods

### Study population and design

This study presents the results from two different cohorts of adult-onset asthma patients: Seinäjoki Adult Asthma Study (SAAS) –cohort (Finland), and Cohort for Reality and Evolution of Adult Asthma in Korea (COREA, Korea). Results are presented separately, but in a similar way. Patients in both cohorts are divided into three different age groups: 1) < 40 years, 2) 40–59.9 years, and 3) ≥ 60 years at asthma diagnosis, respectively.

### Seinäjoki adult asthma study (SAAS)

In Seinäjoki Adult Asthma Study (SAAS), 257 patients (≥15 years of age) were diagnosed with new-onset adult asthma during the years 1999–2002 in Seinäjoki Central Hospital, Finland. Diagnosis of asthma was made by respiratory physician, as previously described [9, 21–24]. Majority of the patients were therapy naïve at baseline. Protocol, and the exclusion and inclusion criteria of SAAS have been previously published [21]. A written informed consent was obtained from all patients, and the study protocol was approved by the Ethics committee of Tampere University Hospital, Tampere, Finland (R12122).

In SAAS-cohort, objective lung function measurements were performed on every patient and the diagnosis was based on significant reversibility/variability in obstruction of the airway. BDR of at least 200 mL and 15% from baseline value (after inhalation of 200 μg of salbutamol) was considered diagnostic for asthma but diagnosis could also be based on peak expiratory flow (PEF) monitoring, bronchial obstruction in response to challenge with allergen or exercise, or reversibility of obstruction with steroid therapy [21]. In the present study, all patients (*n* = 245) with bronchodilator test performed at the time of diagnosis are included, and cross-sectional data from the diagnostic visit is used. Finnish reference values of spirometry were used [25].

### Cohort for reality and evolution of adult asthma Korea (COREA)

The Cohort for Reality and Evolution of Adult Asthma (COREA) is the first asthma cohort in South Korea since 2005 [16, 26–31]. Patients (aged ≥15 years) diagnosed with asthma by allergists or pulmonologists from 21 centers in diverse areas of Korea were enrolled to the study. In COREA, inclusion criteria were a diagnosis of asthma based on clinical symptoms and either a positive bronchodilator test (200 μg of salbutamol) or airway hyperresponsiveness (PC_20_ FEV_1_ ≤ 25 mg/ml methacholine). All enrolled participants signed informed consent. The protocol and design of this cohort were approved by the institutional review board of each center. Of the original 4846 asthma patients in COREA cohort, our study selected a total of 785 patients who were steroid naïve, and had a bronchodilator test performed at the time of diagnosis. In COREA cohort generally, diagnosis of asthma was based on patients having either BDR of at least 200 mL and 12% in spirometry, or at least moderate bronchial hyperreactivity. Majority of the diagnoses in COREA cohort were based on methacholine challenge test.

In both cohorts, smoking status and history were assessed and smoked pack-years (20 cigarettes per day for 1 year) were evaluated. Levels of blood eosinophils and immunoglobulin E (IgE) were measured, skin prick tests were performed, and the use of steroid medication was recorded by a structured questionnaire.

### Statistical analyses

Statistical analyses were performed using SPSS software, version 24 (IBM SPSS, Armonk, NY) or R software, version 3.5.0. Continuous data is expressed as mean ± SD or median and interquartile range, as appropriate. Groups were compared by using one-way ANOVA with Tukey’s post hoc test, Kruskal-Wallis test or χ^2^–test. Correlation analyses were performed by using Spearman’s or Pearson’s correlation tests. A *p-*value < 0.05 was regarded as statistically significant.

## Results

### Clinical characteristics by age in SAAS cohort

There were no differences in gender distribution between the 3 groups with different age at asthma diagnosis, but BMI increased by age (Table 1). Majority of patients in all age groups were never smokers, and the proportion of current smokers decreased with age. As expected, the number of pack-years (among ex and current smokers) increased by age, being highest in the oldest group. Majority of the patients were therapy naïve at the diagnosis of asthma, with < 9% using steroid medication at that time. Furthermore, there were no differences in the levels of blood eosinophils or immunoglobulin E (IgE) between the groups, although the number of atopic patients was found to be significantly higher in the youngest age group (< 40 years) as compared to the older groups (Table 1).

**Table 1:** Baseline clinical characteristics of the 245 patients included from the SAAS cohort

	Age at asthma diagnosis < 40 years *n* = 83	Age at asthma diagnosis 40–59.9 years *n* = 115	Age at asthma diagnosis ≥ 60 years *n* = 47	*p*-value
Age, years	29.2 ± 7.0	50.6 ± 5.3	68.0 ± 5.3	NA
Gender male	33 (39.8%)	48 (41.7%)	22 (46.8%)	0.733
BMI kg·m^− 2^	25.5 (23.1–30.0)	27.1 (24.3–30.1)	28.7 (26.4–31.6) ^a^	**0.006**
Smoking status				**0.003**
Never smokers	46 (55.4%)	49 (42.6%)	23 (48.9%)	
Ex-smokers	14 (16.9%)	43 (37.4%) ^a^	20 (42.6%) ^a^	
Current smokers	23 (27.7%)	23 (20.0%)	4 (8.5%) ^a^	
Pack-years (of ex/current smokers)	5 (3–18)	15 (7–20) ^a^	24 (10–38) ^a,b^	**< 0.001**
Steroid medication in use	4 (4.9%)	10 (8.7%)	4 (8.5%)	0.582
B-eosinophils ×10^9^/L	0.30 (0.19–0.46)	0.22 (0.16–0.40)	0.24 (0.18–0.45)	0.341
IgE kU/L ^c^	98 (38–237)	75 (28–145)	71 (21–138)	0.108
Skin prick positive	41 (54.7%)	30 (29.1%) ^a^	6 (14.6%) ^a^	**< 0.001**

Data is shown as n (%), mean ± SD, or median (interquartile range). *NA* not analyzed, *BMI* body mass index, *B* blood, *IgE* immunoglobulin E

^a^: as compared to group: Age at asthma diagnosis < 40 years *p* < 0.05

^b^ as compared to group: Age at asthma diagnosis 40–59.9 years *p* < 0.05

^c^: data available on 187 patients

### Clinical characteristics by age in the COREA cohort

Patients with asthma onset ≥60 years were more often males, and BMI increased with increasing age of asthma diagnosis (Table 2). Majority of patients in the two groups with asthma diagnosis before 60 years of age were never smokers, but in the oldest group (≥60 years) most patients were ex-smokers. Number of smoked pack-years increased with age at diagnosis, as expected. Blood eosinophil levels and prevalence of atopy were the highest among patients with youngest age at diagnosis of asthma (Table 2). No differences in IgE levels were found between the groups of different age at diagnosis of asthma (Table 2). All patients included from the COREA cohort were steroid-naïve at the diagnosis of asthma.

**Table 2:** Baseline clinical characteristics of the 785 patients included from the COREA cohort

	Age at asthma diagnosis < 40 years *n* = 245	Age at asthma diagnosis 40–59.9 years *n* = 316	Age at asthma diagnosis ≥ 60 years *n* = 224	*p*-value
Age, years	36.3 ± 11.6	54.4 ± 7.8	68.9 ± 5.3	NA
Gender male	103 (42.0%)	141 (44.6%)	118 (52.7%)	0.055
BMI kg·m^−2^	23.3 ± 3.6	24.6 ± 3.5 ^a^	24.6 ± 3.1 ^a^	**< 0.001**
Smoking status				
never smokers	116 (48.5%)	171 (55.2%)	94 (42.9%)	**< 0.001**
ex-smokers	83 (34.3%)	100 (31.3%)	106 (48.4%)	
current smokers	41 (17.2%)	42 (13.6%)	19 (8.7%)	
Pack-years	4 ± 9	9 ± 16 ^a^	17 ± 24 ^a,b^	**< 0.001**
B-eosinophils ×10^9^/L	0.44 ± 0.40	0.33 ± 0.32 ^a^	0.28 ± 0.26 ^a^	**< 0.001**
IgE kU/L ^c^	422 ± 568	320 ± 576	378 ± 650	0.305
Skin prick positive ^d^	115 (64.6%)	85 (47.0%)	18 (19.2%)	**< 0.001**

Data is shown as n (%) and mean ± SD. *NA* not analyzed, *BMI* body mass index, *B* blood, *IgE* immunoglobulin E

^a^: as compared to group: Age at asthma diagnosis < 40 years *p* < 0.05

^b^ as compared to group: Age at asthma diagnosis 40–59.9 years *p *< 0.05.

^c^: data available on 461 patients. ^d^: data available on 463 patients

### Lung function by age in cohorts of SAAS and COREA

In both cohorts, lung function as measured in liters and percentages of predicted value at the time of diagnosis was found to decrease by age (Table 3). In addition, the severity of obstruction, as measured by FEV_1_/FVC ratio, increased by age. In contrast, no differences were found between the groups in the diffusing capacity values, which were measured only in the SAAS-cohort (Table 3). Both the cohorts of SAAS and COREA included also smoking patients (ex or current) and therefore some patients could be considered as having ACO. The proportion of possible ACO patients, i.e. subjects with smoking history of ≥10 pack-years and post-bronchodilator FEV_1_/FVC < 0.7, increased by age. Of the patients in the oldest groups, 22% in the SAAS cohort and 37% in the COREA cohort fulfilled the ACO criteria (Table 3).

**Table 3:** Lung function and prevalence of ACO in cohorts of SAAS and COREA

	Age at asthma diagnosis < 40 years	Age at asthma diagnosis 40–59.9 years	Age at asthma diagnosis ≥ 60 years	*p*-value
**SAAS cohort**				
FEV_1_ L post BD	3.34 (2.90–4.17)	2.87 (2.40–3.36) ^a^	2.01 (1.75–2.50) ^a,b^	**< 0.001**
FEV_1_% pred post BD	90 (84–100)	86 (74–99)	79 (60–89) ^a,b^	**< 0.001**
FEV_1_/FVC post BD	0.81 (0.75–0.87)	0.78 (0.73–0.83) ^a^	0.73 (0.62–0.79) ^a,b^	**< 0.001**
FVC % pred post BD	95 (88–103)	92 (78–103)	87 (73–98) ^a^	**0.012**
DLco % predicted^c^	100 ± 20	95 ± 19	92 ± 18	0.093
DL/VA % predicted^c^	104 ± 19	98 ± 19	97 ± 15	0.106
ACO^d^	3 (3.7%)	11 (9.7%)	10 (22.2%) ^a^	**0.004**
**COREA cohort**				
FEV_1_ L post BD	2.74 ± 0.90	2.23 ± 0.69 ^a^	1.70 ± 0.57 ^a,b^	**< 0.001**
FEV_1_% pred post BD	84 ± 21	82 ± 23	75 ± 24 ^a,b^	**< 0.001**
FEV_1_/FVC post BD	0.77 ± 0.13	0.72 ± 0.13 ^a^	0.66 ± 0.15 ^a,b^	**< 0.001**
FVC% pred post BD	90 ± 16	90 ± 17	84 ± 21 ^a,b^	**< 0.001**
ACO^d^	17 (6.9%)	55 (17.4%)	82 (36.6%)	**< 0.001**

Data is shown as n (%), mean ± SD or median (interquartile range). *DLco* Diffusing capacity of the lung for carbon monoxide, *VA* Alveolar volume

^a^: as compared to group: Age at asthma diagnosis < 40 years *p* < 0.05

^b^ as compared to group: Age at asthma diagnosis 40–59.9 years *p* < 0.05

^c^ Data available from 64 (77.1%), 86 (74.8%) and 33 (70.2%) of patients, respectively

^d^ACO: post BD FEV_1_/FVC < 0.7 and pack-years ≥10

### Bronchodilator response by age in SAAS cohort

Bronchodilator reversibility in FEV_1_ (absolute change in mL, and change in % from the baseline value) was measured at the time of asthma diagnosis in every patient included in the analysis. No significant differences were found between the age groups in FEV_1_ BDR measured either as mL or percentages (Table 4). In addition, the proportion of patients having high reversibility of obstruction (> 400 mL in FEV_1_) did not differ between the age groups (Table 4). The findings remained the same even after exclusion of possible ACO patients (Supplementary Table S1). Furthermore, there were no differences between the age groups in the proportions of patients who fulfilled the reversibility criteria of 200 mL, 12% or both (Table 4).

**Table 4:** Bronchodilator response in FEV_1_ at asthma diagnosis by age groups in SAAS cohort

	Age at asthma diagnosis < 40 years *n* = 83	Age at asthma diagnosis 40–59.9 years *n* = 115	Age at asthma diagnosis ≥ 60 years *n* = 47	*p*-value
FEV_1_ BDR mL	190 (100–330)	130 (60–340)	180 (30–310)	0.266
FEV_1_ BDR %	6.1 (3.1–11.2)	5.5 (1.9–12.1)	8.9 (2.1–20.6)	0.293
Patients with > 400 mL BDR in FEV_1_	15 (18.1%)	23 (20.0%)	6 (12.8%)	0.553
Patients with ≥200 mL BDR in FEV_1_	41 (49.4%)	46 (40.0%)	20 (42.6%)	0.415
Patients with ≥12% BDR in FEV_1_	18 (21.7%)	29 (25.2%)	18 (38.3%)	0.109
Patients with ≥200 mL and 12% BDR in FEV_1_	18 (21.7%)	29 (25.2%)	17 (36.2%)	0.187

Data is shown as n (%) or median (interquartile range). *BDR* bronchodilator response

### Bronchodilator response by age in COREA cohort

Bronchodilator reversibility was higher in patients with younger age at diagnosis when measured as absolute change (mL) in FEV_1_, but not when measured as % change from the baseline value (Table 5). Percentage of patients with absolute (either > 400 mL or ≥ 200 mL) change in FEV_1_ was the highest in the youngest group (< 40 years). However, no differences were found between the groups in the proportions of patients who fulfilled ≥12%, or ≥ 12% and ≥ 200 mL of BDR in FEV_1_ (Table 5). After exclusion of possible ACO patients, BDR in FEV_1_ did not differ between the age groups either in mL or in %, and proportion of patients with > 400 mL BDR in FEV_1_ decreased with age (Supplementary Table S2).

**Table 5:** Bronchodilator response in FEV_1_ at asthma diagnosis by age groups in COREA cohort

	Age at asthma diagnosis < 40 years *n* = 245	Age at asthma diagnosis 40–59.9 years *n* = 316	Age at asthma diagnosis ≥ 60 years *n* = 224	*p*-value
FEV_1_ BDR mL	153 ± 268	139 ± 192	101 ± 175 ^a^	**< 0.001**
FEV_1_ BDR %	7.9 ± 14.1	8.4 ± 12.8	8.6 ± 13.9	0.631
Patients with > 400 mL BDR in FEV_1_	37 (15.1%)	20 (6.3%) ^a^	9 (4.0%) ^a^	**< 0.001**
Patients with ≥200 mL BDR in FEV_1_	95 (38.8%)	104 (32.9%)	49 (21.9%) ^a,b^	**< 0.001**
Patients with ≥12% BDR in FEV_1_	58 (23.7%)	94 (29.8%)	73 (32.6%)	0.088
Patients with ≥200 mL and 12% BDR in FEV_1_	55 (22.5%)	79 (25.0%)	46 (20.5%)	0.467

Data is shown as n (%) and mean ± SD.

^a^: as compared to group: Age at asthma diagnosis < 40 years* p *< 0.05

^b^ as compared to group: Age at asthma diagnosis 40–59.9 years *p* < 0.05

### Correlation between age at asthma diagnosis and bronchodilator response

**Fig. 1:**
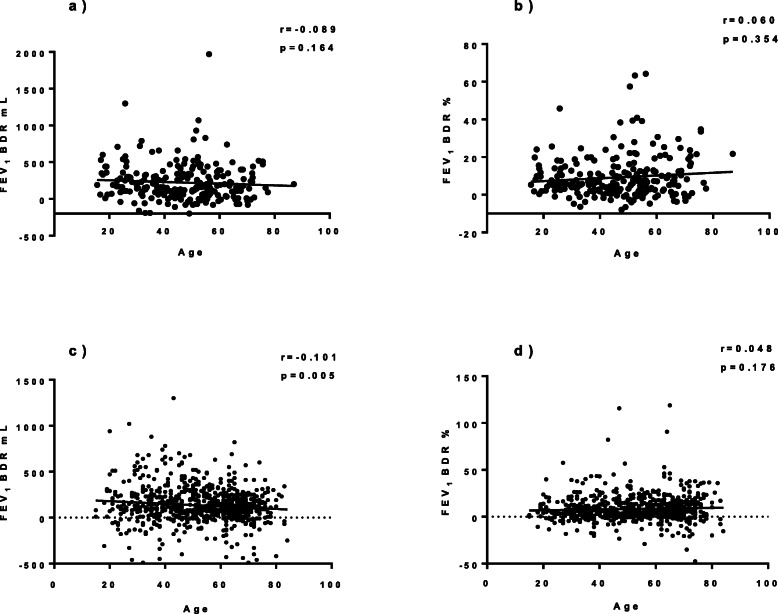
Correlations between age at diagnosis of adult-onset asthma and bronchodilator reversibility in FEV_1_**a**) in mL in SAAS cohort (Spearman’s test), **b**) in percentages in SAAS cohort (Spearman’s test), **c**) in mL in COREA cohort (Pearson’s test), **d**) in percentages in COREA cohort (Pearson’s test). One outlier removed from c) and d). FEV_1_ = Forced expiratory volume in one second, BDR = bronchodilator response

To further evaluate the connection between age at asthma diagnosis and bronchial reversibility, correlations were analyzed. No correlation was found between BDR in FEV_1_ in % and age at asthma diagnosis in either of the cohorts (Fig. 1 b and d). Age at asthma diagnosis and FEV_1_ BDR in mL showed statistically, but not clinically, significant negative correlation (i.e. higher reversibility in younger subjects) in COREA cohort (Fig. 1c), but not in SAAS cohort (Fig. 1a).

## Discussion

We present here the results on bronchodilator response in patients at the time of diagnosis of adult-onset asthma, as measured in two different, carefully described, clinical cohorts of asthma: the Seinäjoki Adult Asthma Study (SAAS) and the Cohort for Reality and Evolution of Adult Asthma in Korea (COREA). BDR as measured in % in FEV_1_ was shown to be similar in different ages of asthma diagnosis. In addition, the majority of patients aged 40 years or older, did not reach the BDR limit of 400 mL in FEV_1_ at the diagnostic time of asthma.

As previously shown, lung function decreased with age in both cohorts [32–35]. BDR has been previously proposed to decrease with age among general population and healthy persons [32, 33, 36]. A study of Quanjer et al. evaluated the change in FEV_1_ after bronchodilator on obstructive clinical patients (asthma, COPD or ACO), and showed association of BDR with age, height, sex and level of respiratory impairment [36]. The change in FEV_1_ was suggested to decline with age, becoming even negative after 50 years of age [36]. Another recent population study on subjects with treated asthma and COPD reported a very limited value of reversibility testing in distinguishing asthma from COPD [37]. In addition, a recent review on BDR in asthma diagnostics stated that the change in FEV_1_ after bronchodilator may not be very sensitive tool in asthma diagnostics, and the sensitivity or specificity of any cut-off levels have not been clearly shown [3]. Our study on adult-onset asthma patients showed the relative response to bronchodilator in FEV_1_ (% from baseline) to be similar despite the age at diagnosis of asthma, and absolute BDR in FEV_1_ (mL) to decrease with increasing age of asthma diagnosis. In keeping with the previous studies, our results thus showed, that BDR in FEV_1_ does not increase after age of 40 years. In addition, correlation analyses between age and BDR in FEV_1_ did not show clinically meaningful correlation, further indicating that BDR remains stable despite increasing age of asthma onset.

Increasing evidence shows that asthma starting at adult age is very common [14–16]. As compared with child-onset disease, adult-onset asthma patients are less often allergic and have poorer prognosis with low remission rate [24, 38]. At adult age the differential diagnostics between asthma, ACO and COPD becomes essential, because misdiagnosing adult smoking patients’ asthma or ACO for COPD may lead to severe morbidity on individual level. However, widely accepted diagnostic criteria for ACO are still missing.

Several COPD guidelines have presented suggestions for ACO criteria [10–12]. Major proposed criteria for ACO among population with COPD have been a significant BDR in FEV_1_ (> 15% and > 400 mL), sputum eosinophilia and elevated levels of exhaled nitric oxide (FeNO) [10–12]. Further proposal for ACO criteria has included an age cut-off of 40 years: asthma should be diagnosed earlier, or high reversibility in FEV_1_ > 400 mL should be present [7]. There are, however, different pathways in developing asthma-COPD overlap. The most studied perspective is when a patient has a previous diagnosis of COPD and develops ACO afterwards; a viewpoint widely reflected in the previous guidelines and suggestions for ACO criteria [7, 10–12]. However, ACO may also become diagnosed in patients with previous asthma or, more importantly, in patients without any previous diagnoses. This perspective is only remotely studied, even though the implementation challenges of the previously suggested ACO criteria among general population have already been discussed [13].

Our results showed, that BDR % in FEV_1_ does not change with age, and even fewer asthma patients have > 400 mL of BDR in FEV_1_ at the diagnostic point when age of asthma-onset increases. As partial reversibility of the obstruction is also a feature of COPD, the suggested limit of > 400 mL BDR in FEV_1_ for asthma-COPD overlap diagnosis after 40 years of age would presumably reduce the overuse of inhaled corticosteroids. The high BDR cut-off would improve specificity, but on the cost of sensitivity. In practice, this means that a majority of subjects with new onset adult asthma as component of their ACO would have to fulfil this strict criterion of reversibility. In our study, of the patients aged 40 years or older at the time of asthma diagnosis, only 5% in COREA cohort and 18% in SAAS cohort fulfilled the limit of BDR > 400 mL in FEV_1_. That is to say, 82–95% of the adult-onset asthma patients do not reach the limit of BDR > 400 mL in FEV_1_. In addition, atopy was shown to decrease with age, in keeping with previous studies [39]. Thus, if using the suggested > 400 mL limit in non-atopic patients for asthma-COPD overlap diagnosis, most adult-onset ACO diagnoses would be missed.

In COPD it has been shown that BDR in FEV_1_ decreases with increasing severity of COPD [4, 5]. In our study, some patients with smoking history ≥10 pack-years and post BD FEV_1_/FVC < 0.7 could be considered as having asthma-COPD overlap, although the spirometry was measured before the start of the asthma therapy. To avoid bias caused by possible ACO patients having presumably lower response to bronchodilator, we further performed analyses with exclusion of possible ACO patients. The main result remained the same, and thus, our finding is not biased by ACO or COPD.

Major strength of the current study is that we have two large, well defined, real-world cohorts of adult-onset asthma, altogether a study population reflecting clinical reality exceptionally well. The large number of enrolled patients enables us to examine the BDR at the moment of asthma diagnosis in patients over the whole adult-age span, without losing power in analyses. Patients with smoking history are included in the study cohorts, and smoking intensity of the patients is well described. In accordance to the guidelines, the diagnosis of asthma was based on clinical history and objective lung function measurements, and bronchodilator test was measured in every patient. In SAAS and COREA cohorts, however, the diagnostic practices differ slightly from one another. In some patients, the diagnosis of asthma was made based on other objective lung function measurements than positive bronchodilation test, leading to somewhat lower BDR results. This could be considered as a limitation. Despite this, the level of change in FEV_1_ after administration of a bronchodilator was similar in these cohorts and the results of both cohorts are in line, increasing the reliability of our results. The reversibility status of individual patients has been shown to vary over time [4, 5]. Thus, another limitation of our study could be that only the BDR at the diagnostic point of asthma was evaluated. However, in our study, most patients were steroid naïve at the diagnostic visit and inhaled corticosteroid medication was started after diagnostic measures. Therefore, evaluating BDR in several time points in our study would not have been informative.

## Conclusions

In conclusion, we have shown that the BDR in FEV_1_ at asthma diagnosis is constant over large age span range in adult-onset asthma. In addition, minority of patients with adult-onset asthma have > 400 mL BDR in FEV_1_ at time of diagnosis. These findings are to be considered when designing diagnostic guidelines concerning asthma starting at adult age, including asthma-COPD overlap.

## Supplementary information

**Additional file 1: Table S1.** Bronchodilator response (BDR) in FEV_1_ grouped by age at asthma diagnosis in SAAS cohort after exclusion of ACO patients. **Table S2.** Bronchodilator response (BDR) in FEV_1_ grouped by age at asthma diagnosis in COREA cohort after exclusion of ACO patients.

## Data Availability

All data generated or analyzed during this study are included in this published article (and its Supplementary File). According to ethical permission and patient data-protection laws of Finland, single patient data cannot be made available, but aggregated data is available from authors on reasonable request.

## Abbreviations

FEV_1_: Forced expiratory volume in one second
FVC: Forced vital capacity
COPD: Chronic obstructive pulmonary disease
ACO: Asthma-COPD overlap
BDR: Bronchodilator response
SAAS: Seinäjoki Adult Asthma Study
COREA: Cohort for Reality and Evolution of Adult Asthma in Korea
PEF: Peak expiratory flow
PC_20_FEV_1_: Provocative concentration causing a 20% fall in forced expiratory volume in one second
IgE: Immunoglobulin E
BMI: Body mass index
DLco: Diffusing capacity of the lung for carbon monoxide
DL/VA: Diffusing capacity of the lung/ alveolar volume
