# Supplementary material for: Relationship between age and bronchodilator response at diagnosis in adult-onset asthma
Source: Respir Res. 2020 Jul 13;21:179. doi: 10.1186/s12931-020-01441-w (PMC7359254; doi:10.1186/s12931-020-01441-w)
Supplement: Supplementary file 1 — Additional file 1: Table S1. Bronchodilator response (BDR) in FEV1 grouped by age at asthma diagnosis in SAAS cohort after exclusion of ACO patients. Table S2. Bronchodilator response (BDR) in FEV1 grouped by age at asthma diagnosis in COREA cohort after exclusion of ACO patients. [file 12931_2020_1441_MOESM1_ESM.docx]

**Supplementary material**

**Relationship between age and bronchodilator response at diagnosis in adult-onset asthma**

Minna Tommola, MD, Ha-Kyeong Won, MD, Pinja Ilmarinen, PhD, Heewon Jung, MS, Leena E. Tuomisto, MD, PhD, Lauri Lehtimäki, MD, PhD, Onni Niemelä, MD, PhD, Tae-Bum Kim, MD, PhD, Hannu Kankaanranta, MD, PhD

Table S1. Bronchodilator response (BDR) in FEV_1_ grouped by age at asthma diagnosis in SAAS cohort after exclusion of ACO patients

|  | Age at asthma diagnosis  < 40 years  n=79 | Age at asthma diagnosis  40-59.9 years  n=102 | Age at asthma diagnosis  ≥ 60 years  n=35 | p-value |
| --- | --- | --- | --- | --- |
| FEV_1_ BDR mL | 190 (100-350) | 125 (50-330) | 160 (20-290) | 0.168 |
| FEV_1_ BDR % | 5.9 (2.7-10.9) | 5.1 (1.9-11.3) | 7.3 (0.9-19.5) | 0.366 |
| Patients with >400mL BDR | 15 (19.0%) | 17 (16.7%) | 5 (14.3%) | 0.816 |

Data is shown as n (%) or median (interquartile range)

Table S2. Bronchodilator response (BDR) in FEV_1_ grouped by age at asthma diagnosis in COREA cohort after exclusion of ACO patients

|  | Age at asthma diagnosis  < 40 years  n=228 | Age at asthma diagnosis  40-59.9 years  n=261 | Age at asthma diagnosis  ≥ 60 years  n=142 | p-value |
| --- | --- | --- | --- | --- |
| FEV_1_ BDR mL | 142.98 ± 268.08 | 134.75 ±184.35 | 95.7 ± 151.61 | 0.098 |
| FEV_1_ BDR % | 6.8 ± 11.9 | 7.6 ± 11.2 | 7.7± 12.8 | 0.693 |
| Patients with >400mL BDR | 32 (14.8%) | 13 (5.0%) | 4 (2.8%) | **<0.001** |

Data is shown as n (%) and mean ± SD
